# Etiological and Resistance Profile of Bacteria Involved in Urinary Tract Infections in Young Children

**DOI:** 10.1155/2017/4909452

**Published:** 2017-04-11

**Authors:** Antonio Sorlózano-Puerto, José María Gómez-Luque, Juan de Dios Luna-del-Castillo, José María Navarro-Marí, José Gutiérrez-Fernández

**Affiliations:** ^1^Departamento de Microbiología, Universidad de Granada-ibs.Granada, Granada, Spain; ^2^UGC de Pediatría, Complejo Hospitalario Universitario de Granada-ibs.Granada, Hospital General Virgen de las Nieves, Granada, Spain; ^3^Departamento de Bioestadística, Universidad de Granada-ibs.Granada, Granada, Spain; ^4^Laboratorio de Microbiología, Complejo Hospitalario Universitario de Granada-ibs.Granada, Hospital General Virgen de las Nieves, Granada, Spain

## Abstract

*Background.* The objective of this study was to identify the bacteria most frequently responsible for urinary tract infection (UTI) in the population of under-2-year-olds in our geographic area and to evaluate the activity of antibiotics widely used for UTI treatment during a 4-year study period.* Materials and Methods.* A retrospective analysis was conducted of data on the identification and susceptibility of microorganisms isolated in urine samples from children under 2 years of age.* Results.* A total of 1,045 uropathogens were isolated.* Escherichia coli* accounted for the majority (60.3%) of these, followed by* Enterococcus faecalis* (22.4%) and* Klebsiella* spp. (6.5%). The highest* E. coli* susceptibility rates (>90%) were to piperacillin-tazobactam, cefuroxime, cefotaxime, ceftazidime, imipenem, gentamicin, nitrofurantoin, and fosfomycin, and the lowest were to amoxicillin-clavulanic acid and cotrimoxazole. Among all bacteria isolated, we highlight the overall high activity of piperacillin-tazobactam, imipenem, nitrofurantoin, and fosfomycin against both community and hospital isolates and the reduced activity of amoxicillin-clavulanic acid, cephalosporins, gentamicin, and cotrimoxazole. There was no significant change in the total activity of any of the studied antibiotics over the 4-year study period.* Conclusion.* Empiric treatment with amoxicillin-clavulanic acid, cotrimoxazole, cephalosporins, and gentamicin may be inadequate due to their limited activity against uropathogens in our setting.

## 1. Introduction

Urinary tract infections (UTIs) are frequent during childhood, with a prevalence of 2–9% depending on the age and gender. The specificity of clinical manifestations decreases with younger age, and around 5–8% of under-2-year-olds may present fever as sole UTI symptom [1]. Febrile UTI in this age group can be associated with permanent renal lesions, and bacterial infection of the kidney is detected by cortical imaging in 50–85% of children with a first febrile UTI, regardless of the presence of major urinary tract abnormalities [2]. It is therefore important to obtain a precise clinical and etiological diagnosis, start early and effective treatment, and document the integrity of the urinary tract in order to avoid recurrent infections, complications (e.g., renal scarring), and sequelae (e.g., hypertension and chronic renal disease) [3–5].

In both infants and adults, the main causative agent of UTI is* Escherichia coli*, whose antibiotic susceptibility pattern is the first to be examined in selecting empiric antibiotic treatment. Other fecal bacteria that occasionally cause UTI are* Klebsiella* spp.,* Proteus mirabilis*, other Gram-negative enteric bacilli, and enterococci [6].

Depending on the age of the patient and the severity of the disease, published guidelines recommend parenteral antibiotics followed by oral antibiotics for the initial treatment of infant UTI [3, 7, 8]. Effective empiric treatment requires knowledge of the microorganisms involved and awareness of the antibiotic susceptibility patterns of the most frequent uropathogens in each geographic setting and their evolution [9–11]. Consequently, the objectives of the present study were to identify the bacteria most frequently responsible for UTI in the pediatric population aged under 2 years in our care setting, to determine their susceptibility to the antibiotics habitually used in this population, and to evaluate the activity against all isolates of antibiotics widely used for the treatment of community- and hospital-acquired UTIs.

## 2. Methods

The methodology used in this study was previously reported by our group [10]. A retrospective analysis was conducted of laboratory data routinely collected by the Microbiology Department of Granada University Hospital Complex (southern Spain) on the identification and susceptibility of all consecutive bacteria isolated from urine samples of children under 2 years of age with a first-time symptomatic, culture-verified UTI diagnosed between January 2011 and December 2014.

The study included samples obtained by midstream clean-catch, bladder catheterization, or suprapubic bladder aspiration. The microbiological diagnosis of UTI required ≥10^5^ colony-forming units (CFU)/mL of a single microorganism in midstream, or ≥10^4^ CFU/mL of a single microorganism in catheter samples, or any bacterial growth in urine obtained by suprapubic bladder aspiration. Urine cultures with growth of more than two microorganisms were considered contaminated.

The following samples were excluded: (i) urine samples obtained by plastic bag applied to the perineum, only valid when the result is negative; (ii) samples from previously hospitalized individuals; (iii) those from individuals who had received antibiotic therapy during the 6 months before the UTI episode, and (iv) duplicate positive urine cultures (i.e., of the same genus or species obtained sequentially from the same UTI episode). Furthermore, if a patient provided more than one urine sample, regardless of the origin (community or hospital), an interval of at least 6 months had to elapse before the second sample was included in the study.

The MicroScan system (Siemens Healthcare Diagnostics, Madrid, Spain) was used for identification and susceptibility profiling of all bacteria from positive urine cultures. Amoxicillin-clavulanic acid, piperacillin-tazobactam, cefuroxime, cefotaxime, ceftazidime, imipenem, gentamicin, cotrimoxazole, nitrofurantoin, and fosfomycin were tested against* Enterobacteriaceae*. Piperacillin-tazobactam, ceftazidime, imipenem, and gentamicin were tested against nonfermenting Gram-negative bacilli. Ampicillin, nitrofurantoin, and fosfomycin were tested against* Enterococcus* spp.

Isolates were categorized as susceptible, intermediate, or resistant to each antibiotic according to Clinical and Laboratory Standards Institute (CLSI) classifications [12]. The clinical categorization of all isolates against nitrofurantoin followed the recommendations of the European Committee on Antimicrobial Susceptibility Testing (EUCAST) [13]. For each study year and each bacterial species identified, the proportion of susceptible organisms was calculated by dividing the number of urinary isolates susceptible to each antibiotic by the number of organisms tested against that antimicrobial agent, grouping together intermediately resistant and resistant organisms.

We evaluated the activity of each antibiotic against all bacteria isolated during the 4-year study period, making the following assumptions: (1) Each of the aforementioned antibiotics is potentially active against enterobacteria; (2) amoxicillin-clavulanic acid, cefuroxime, cefotaxime, nitrofurantoin, and fosfomycin have no activity against nonfermenting Gram-negative bacilli, and* Pseudomonas aeruginosa* is intrinsically resistant to cotrimoxazole; (3) among enterococci, the response to ampicillin predicts the response to amoxicillin-clavulanic acid, piperacillin-tazobactam, and imipenem, while cefuroxime, cefotaxime, ceftazidime, gentamicin, and cotrimoxazole are not clinically active against these microorganisms [14]; (4) the activity of fosfomycin against enterobacteria and enterococci can be assessed by using the cutoff points recommended by the CLSI for this antibiotic against* E. coli* and* Enterococcus faecalis*, respectively [15, 16]; and (5) the activity of nitrofurantoin against enterobacteria can be assessed by using the cutoff points recommended by the EUCAST for this antibiotic in* E. coli*.

A descriptive statistical analysis was performed, and differences in susceptibility rates were analyzed using Pearson's *χ*^2^ test and contingency tables with Fisher's exact test. Logistic regression was used to analyze the evolution of antibiotic activity against isolated bacteria in UTIs over the 4-year study period. STATA 13.1 (StataCorp, TX, USA) was used for all analyses. *P* < 0.05 was considered significant in all tests.

### 2.1. Ethics Statements

The study protocol was carried out in accordance with the Declaration of Helsinki and “Commission of Ethics and Health Research of the Hospital Centres and Districts of Healthcare” (*“Comisión de Ética e Investigación Sanitaria de los Centros Hospitalarios y Distritos de Atención Sanitaria”*). This was a noninterventional study involving only routine procedures. Biological material was used solely for standard UTI diagnosis prescribed by physicians, and there was no additional sampling or modification of the standard laboratory protocol. Data analyses were carried out using an anonymous database. For these reasons, no approval was required according to national guidelines. Permission to access and use the data was granted by the Unit for Clinical Management of Infectious Diseases and Clinical Microbiology of the University Hospital Complex of Granada, Spain.

## 3. Results

Over the 4-year study period, the 1,045 bacteria identified included 811 (77.6%) Gram-negative bacilli (including 790* Enterobacteriaceae *and 21* P. aeruginosa*) and 234 (22.4%) Gram-positive cocci (all* E. faecalis*).* E. coli* was the most frequently identified UTI agent in each year (60.3% of all isolates; range, 58.1%–62.1%) in both community (61.1%; range, 59.7%–63.0%) and hospital (57.4%; range, 48.9%–64.7%) isolates, followed by* E. faecalis* (22.4%; range, 18.6%–27.8%) and* Klebsiella* spp. (6.5%; range, 4.8%–8.0%). Other bacteria (*Proteus *spp.,* Enterobacter* spp.,* Citrobacter *spp.,* Serratia marcescens*,* Morganella morganii*, and* P. aeruginosa*) accounted for only 11.3% of community isolates and 9.0% of hospital isolates. No staphylococci or group B streptococci were isolated.

### 3.1. Antibiotic Susceptibility of the Isolated Bacteria

Table 1 exhibits the antimicrobial susceptibilities of the most frequent microorganisms,* E. coli* and* E. faecalis*.* E. coli* showed high susceptibility to piperacillin-tazobactam, cefuroxime, cefotaxime, ceftazidime, imipenem, gentamicin, nitrofurantoin, and fosfomycin, with annual resistance rates <10%; however, resistance to amoxicillin-clavulanic acid, and cotrimoxazole, key antibiotics in the oral treatment of community-acquired UTIs, was recorded in at least 20–30% of* E. coli* isolates in most years. There were no significant differences in total susceptibility between the community and hospital isolates (amoxicillin-clavulanic acid [*p* = 0.910], piperacillin-tazobactam [*p* = 0.060], cefuroxime [*p* = 1.000], cefotaxime [*p* = 0.070], ceftazidime [*p* = 0.074], imipenem [*p* = 1.000], gentamicin [*p* = 1.000], cotrimoxazole [*p* = 0.064], nitrofurantoin [*p* = 1.000], or fosfomycin [*p* = 0.213]).

In general, isolates of* Klebsiella pneumoniae* and* Klebsiella oxytoca* species were highly susceptible, although there was wide variability in the percentages of susceptibility to different antibiotics (range 63–100%), with no significant differences between community and hospital isolates (amoxicillin-clavulanic acid [*p* = 1.000], piperacillin-tazobactam [*p* = 0.672], cefuroxime [*p* = 1.000], cefotaxime [*p* = 0.533], ceftazidime [*p* = 0.120], imipenem [*p* = 1.000], gentamicin [*p* = 1.000], cotrimoxazole [*p* = 0.579], nitrofurantoin [*p* = 0.334], and fosfomycin [*p* = 0.276]).

The incidence of ESBL-producing* E. coli* or* Klebsiella* spp. among community and hospital isolates was very low (5 isolates in 2012, 1 in 2013, and 6 in 2014); hence, their influence on the activity of beta-lactam antibiotics (mainly the cephalosporins studied) was also low.

Other* Enterobacteriaceae* represented 10.1% of community isolates and 4.5% of hospital isolates. Considering all species together, the percentages of susceptibility to the different study antibiotics were highly variable in the different years, between 8% to nitrofurantoin and 100% to some beta-lactam antibiotics (piperacillin-tazobactam and cephalosporins). This group showed high susceptibility to piperacillin-tazobactam, cefuroxime, cefotaxime, and ceftazidime, with annual resistance rates <10%, whereas resistance to amoxicillin-clavulanic acid, imipenem, gentamicin, cotrimoxazole, nitrofurantoin, and fosfomycin was recorded in at least 20–30% of isolates in most years. The resistance rates of these bacteria to amoxicillin-clavulanic acid were higher in hospital isolates (*p* = 0.011), but there was no difference between community and hospital isolates in resistance rates to any other antibiotic.

*P. aeruginosa* exhibited high susceptibility to piperacillin-tazobactam, ceftazidime, imipenem, and gentamicin, with no significant differences between community and hospital isolates. However, the reduced number of isolates during the 4-year study period (21 isolates; 1.2% of community isolates and 4.5% of hospital isolates) prevents the drawing of meaningful comparisons.

As shown in Table 1, we highlight the elevated frequency of* E. faecalis* and its high rates of susceptibility to ampicillin (100%), nitrofurantoin (94–100%), and fosfomycin (94–100%) in both community and hospital isolates, with no significant differences between them (*p* = 1.000).

### 3.2. Evolution of Antibiotic Activity against Isolated Bacteria in UTIs

**Figure 1:**
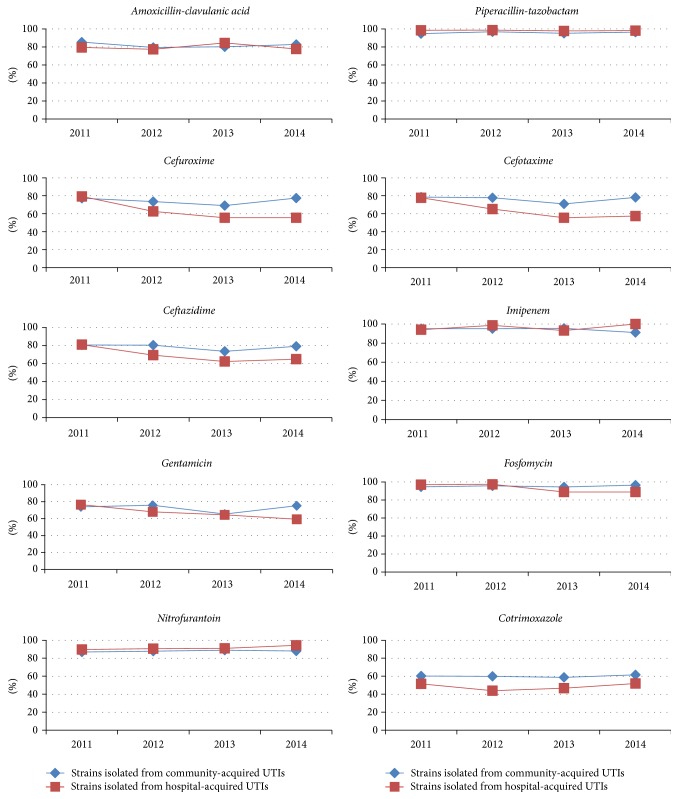
Activity (in %) of the different antibiotics against all bacteria isolated in urine samples during 2011–2014.

Figure 1 depicts the activity of the tested antibiotics against the 1,045 bacteria isolated in urine during the 4-year study period. The activity of cefuroxime (*p* = 0.002), cefotaxime (*p* = 0.001), ceftazidime (*p* = 0.009), and cotrimoxazole (*p* = 0.001) was significantly higher in community-origin than in hospital-origin isolates; however, amoxicillin-clavulanic acid (*p* = 0.346), piperacillin-tazobactam (*p* = 0.176), imipenem (*p* = 0.144), gentamicin (*p* = 0.144), fosfomycin (*p* = 0.314), and nitrofurantoin (*p* = 0.164) showed no difference in activity between community- and hospital-origin isolates.

There was no significant change in the total activity of any of the studied antibiotics over the 4-year study period (amoxicillin-clavulanic acid [*p* = 0.624], piperacillin-tazobactam [*p* = 0.945], cefuroxime [*p* = 0.192], cefotaxime [*p* = 0.124], ceftazidime [*p* = 0.131], imipenem [*p* = 0.161], gentamicin [*p* = 0.274], fosfomycin [*p* = 0.490], nitrofurantoin [*p* = 0.462], or cotrimoxazole [*p* = 0.869]). However, the activity of cefuroxime, cefotaxime, ceftazidime, and gentamicin against hospital-origin isolates significantly decreased (*p* < 0.001), as shown in Figure 1.

## 4. Discussion

Our research group previously published a retrospective analysis on the identification and susceptibility pattern of all bacteria isolated from urine samples with microbiological confirmation of UTI in the Microbiology Department of Granada University Hospital Complex (Granada, Spain) between January 2006 and December 2012 [10]. This hospital complex meets the demands of the North Hospital Area of Granada province, serving a reference population of around 440,000 inhabitants. Within this geographic area, we compared data on 1,045 urine samples from the pediatric population (under 2 years of age) with the previously reported data on 31,758 samples, mainly from individuals over 14 years of age.

In both cases,* E. coli* was the most frequently bacterium isolated in urine samples of patients with UTI (60.3% in pediatric population versus 55.2% in adult population), followed by* E. faecalis* (22.4% versus 18.0%). The isolation frequency of the remaining bacteria was also highly similar between the studies. These data are consistent with previous findings that* E. coli* is the most frequently isolated uropathogen at all ages in our region and in other countries [17–21]. The frequency of* Enterococcus* spp. in our setting was higher than that of enterobacteria other than* E. coli*. Therapeutic recommendations must take into account the reduced activity of cephalosporins, aminoglycosides, and cotrimoxazole against* Enterococcus* spp.

In general, the bacteria isolated in young children were more susceptible than those isolated in adults. A high rate of resistance (20–30% of isolates in most years) was found to antibiotics such as amoxicillin-clavulanic acid and cotrimoxazole in* E. coli* and to nitrofurantoin in* P. mirabilis* (main bacteria in the group of other* Enterobacteriaceae*). The low frequency of ESBL-producing enterobacteria (1.1%) and the elevated susceptibility of enterococci to the studied antibiotics would contribute to the higher bacterial susceptibility rates in the children. As observed by various authors, increased resistance rates are associated with greater exposure to antibiotics [4, 22, 23], and the lesser exposure of infants in comparison to adults may also explain the increased susceptibility of UTI isolates in the pediatric population.

In agreement with our results, various Spanish studies of pediatric populations have reported a high resistance of* E. coli* to cotrimoxazole and variable rates of resistance to amoxicillin-clavulanic acid (these antibiotics would therefore not be suitable for empiric UTI treatment) but an elevated susceptibility to second- and third-generation cephalosporins, fosfomycin, nitrofurantoin, and aminoglycosides [9, 24].* Klebsiella* spp. showed a high but widely variable susceptibility to all study antibiotics, as previously reported in our country [9, 24, 25]. However, antibiotic resistance rates were higher for other enterobacteria (including the highly frequent* P. mirabilis*) than for* E. coli*, as reported by some uroculture studies in general and pediatric populations [9, 10, 24].* P. aeruginosa* has classically demonstrated high susceptibility to piperacillin-tazobactam, ceftazidime, carbapenems (mainly imipenem), and aminoglycosides, as in the present study [18, 25, 26]. Finally, although cephalosporins and aminoglycosides are slightly superior against enterobacteria in comparison to amoxicillin-clavulanic acid,* Enterococcus* spp. is intrinsically resistant to these antibiotics. Hence, amoxicillin-clavulanic acid, fosfomycin, or nitrofurantoin can be used against these bacteria, which were highly frequent in our study, although this is not necessarily the case in other geographic areas of our country [25].

Based on different international recommendations, the Spanish Association of Pediatrics has established precise guidelines for the initial empiric treatment of UTIs according to the age of the child. The recommendation in the first three months of life, a period of maximum risk of renal involvement and progression to sepsis, is parenteral treatment with two antibiotics, optimally ampicillin with an aminoglycoside (usually gentamicin) or cefotaxime at doses recommended for neonatal sepsis. After resolution of the clinical manifestations, these can be replaced with oral antibiotic(s) (amoxicillin, with or without clavulanic acid, cotrimoxazole, cefadroxil, or cefixime) for 10–14 days, after examining the pattern of susceptibility of the causative agent to antimicrobials and ruling out any urinary tract obstruction. For children over 3 months of age with moderate or severe febrile UTI and moderate or severe clinical status, initial parenteral treatment with gentamicin or a cephalosporin (cefotaxime, ceftriaxone, or cefuroxime) is recommended; if the symptoms improve, the treatment can be continued for 10–14 days in accordance with the uroculture results and evaluation of the susceptibility of the agent. For children over 3 months of age with febrile UTI but no impairment of their general health status, oral treatment is recommended for 7–10 days; although various agents are valid options, clinical guidelines in our setting recommend cefixime as the first choice and cefuroxime as the second [27].

The activity of any antibiotic used for empiric UTI treatment can be estimated a priori according to the incidence of different pathogens and the accumulated resistance [10]. Some authors consider that an antibiotic with a rate of accumulated bacterial resistance above 15% of isolates should not be used in empiric therapy [9]. In this regard, the antibiotics with highest activity (Figure 1) were piperacillin-tazobactam, imipenem, fosfomycin, and nitrofurantoin, which are not usually selected for empiric UTI treatment in young children. In contrast, the antibiotics that were least active (resistance rates >15% of isolates), that is, amoxicillin-clavulanic acid, second- and third-generation cephalosporins, gentamicin, and cotrimoxazole, are considered first-line antibiotics in the clinical guidelines. As noted above, our findings are explained by the high resistance of enterobacteria, especially* E. coli*, to amoxicillin-clavulanic acid and cotrimoxazole, and by the elevated frequency of* E. faecalis* isolates.

Some researchers found that children with acute pyelonephritis were effectively treated with parenteral or oral doses of amoxicillin-clavulanic acid in an area with a low resistance rate [28]. However, in settings with higher resistance to amoxicillin-clavulanic acid, such as ours, it is appropriate to choose alternative antibiotics in accordance with local susceptibility patterns. In most countries, resistance to cotrimoxazole and aminoglycosides, especially in* Enterococcus* spp. but also in* Enterobacteriaceae*, is now so high that they are no longer suitable for empiric UTI treatment in young children [4, 11, 23]. In their place, cephalosporins have become the empiric drug of choice, but uropathogen resistance to cephalosporins is also increasing [4].

Among alternative options, nitrofurantoin is a bacteriostatic agent for Gram-positive bacteria but a bactericide for Gram-negative bacteria and has been orally administered to treat UTI in children aged over 2-3 years; moreover, the susceptibility of* E. coli* to this drug remains high [4]. Some studies have shown that the renal excretion of nitrofurantoin is adequate, but it is not recommended in complicated UTI because it is a bacteriostatic antibiotic in some cases [5]. However, its oral administration may be an option for the empiric oral treatment of mild nonfebrile and/or localized UTIs (cystitis and urethritis) in children over the age of 2 years. In these cases, another possibility is oral treatment with fosfomycin, an active bactericide antibiotic against numerous Gram-positive and Gram-negative bacteria species; high and prolonged concentrations have been observed in urine, and it has demonstrated effectiveness and good tolerance in young children [29]. It could therefore also be considered in the empiric oral treatment of mild nonfebrile and localized UTIs.

In children, it is exceptional to resort to antibiotics such as carbapenems to cover possible multiresistant microorganisms (e.g., ESBL-producing bacteria) given their low frequency, as also observed in the present study [6]. In general, these antibiotics are not recommended as first-line treatments in children because of the risk of a rapid increase in carbapenemase-producing bacteria [30]. Nevertheless, their administration could be considered in severe cases of febrile UTI in children.

Finally, the main study limitation was the inability to stratify the population under investigation, because the laboratory computer system from which identification and susceptibility data were drawn only gathers the information required to correctly manage the process from request reception to report delivery. Thus, the database does not contain complete information on the sex of patients or on the symptoms (fever, malodorous urine, irritability, poor feeding, vomiting, abdominal distension, etc.) or type (complicated or uncomplicated) of their ITU, among other data. This prevents conclusions being drawn on the relationship between the microorganisms or their resistance/susceptibility rates and the characteristics of the study population.

## 5. Conclusion

In conclusion,* E. coli* was the most frequently isolated bacterium in both community- and hospital-acquired UTIs in our setting, while other bacteria such as* E. faecalis* also represented a large proportion of the etiologic agents detected. Amoxicillin-clavulanic acid, cephalosporins, gentamicin, and cotrimoxazole may be inadequate for the empiric treatment of community or hospital-acquired UTI in children under the age of 2 years, because of the limited activity of these antibiotics against uropathogens in our geographic area. Fosfomycin is an alternative in pediatric patients with uncomplicated UTI, while piperacillin-tazobactam and imipenem are options in more severe UTIs (e.g., neonates, presence of anatomic abnormalities, immunodeficiency, or urinary sepsis). Regular follow-ups are essential in each geographic area to establish local rates of uropathogen resistance to available antibiotics, modifying the protocol for empiric UTI treatment accordingly when changes are detected in the susceptibility profiles of the bacteria.

## Figures and Tables

**Table 1:** Antimicrobial susceptibilities for urinary *Escherichia coli* and *Enterococcus faecalis* by year of isolation.

	*E. coli* susceptibility rate by year in community isolates				*E. coli* susceptibility rate by year in hospital isolates				*E. faecalis* susceptibility rate by year in community isolates				*E. faecalis* susceptibility rate by year in hospital isolates			
	2011 *n* = 123	2012 *n* = 119	2013 *n* = 110	2014 *n* = 139	2011 *n* = 44	2012 *n* = 45	2013 *n* = 22	2014 *n* = 28	2011 *n* = 40	2012 *n* = 35	2013 *n* = 47	2014 *n* = 46	2011 *n* = 11	2012 *n* = 21	2013 *n* = 16	2014 *n* = 18
Ampicillin	—	—	—	—	—	—	—	—	100%	100%	100%	100%	100%	100%	100%	100%
Amoxicillin-clavulanic acid	79%	72%	75%	77%	77%	73%	91%	71%	100%	100%	100%	100%	100%	100%	100%	100%
Piperacillin-tazobactam	93%	96%	94%	96%	98%	98%	100%	100%	100%	100%	100%	100%	100%	100%	100%	100%
Cefuroxime	95%	95%	96%	98%	98%	93%	100%	96%	—	—	—	—	—	—	—	—
Cefotaxime	98%	99%	100%	99%	95%	93%	100%	96%	—	—	—	—	—	—	—	—
Ceftazidime	100%	99%	100%	99%	98%	96%	100%	100%	—	—	—	—	—	—	—	—
Imipenem	98%	100%	99%	100%	98%	100%	100%	100%	100%	100%	100%	100%	100%	100%	100%	100%
Gentamicin	92%	94%	91%	96%	91%	93%	100%	93%	—	—	—	—	—	—	—	—
Cotrimoxazole	73%	74%	81%	75%	59%	60%	82%	82%	—	—	—	—	—	—	—	—
Nitrofurantoin	98%	100%	98%	100%	100%	98%	100%	100%	98%	94%	98%	100%	100%	95%	100%	100%
Fosfomycin	99%	100%	99%	100%	100%	100%	100%	93%	95%	100%	98%	100%	100%	100%	100%	94%
